# Inflammatory Mediator Profiling of *n*-butanol Exposed Upper Airways in Individuals with Multiple Chemical Sensitivity

**DOI:** 10.1371/journal.pone.0143534

**Published:** 2015-11-23

**Authors:** Thomas Meinertz Dantoft, Sine Skovbjerg, Linus Andersson, Anna-Sara Claeson, Nina Lind, Steven Nordin, Susanne Brix

**Affiliations:** 1 Danish Research Centre for Chemical Sensitivities, Copenhagen University Hospital, Gentofte, Denmark; 2 Center for Biological Sequence Analysis, Department of Systems Biology, Technical University of Denmark, Kongens Lyngby, Denmark; 3 Research Centre for Prevention and Health, Capital Region, Copenhagen, Denmark; 4 Department of Psychology, Umeå University, Umeå, Sweden; 5 Department of Occupational and Public Health Sciences, University of Gävle, Umeå, Sweden; Universitatsklinikum Freiburg, GERMANY

## Abstract

**Background:**

Multiple Chemical Sensitivity (MCS) is a chronic condition characterized by reports of recurrent symptoms in response to low level exposure to various chemical substances. Recent findings suggests that dysregulation of the immune system may play a role in MCS pathophysiology.

**Objectives:**

The aim of this study was to examine baseline and low dose *n*-butanol-induced upper airway inflammatory response profiles in MCS subjects versus healthy controls.

**Method:**

Eighteen participants with MCS and 18 age- and sex-matched healthy controls were enrolled in the study. Epithelial lining fluid was collected from the nasal cavity at three time points: baseline, within 15 minutes after being exposed to 3.7 ppm *n*-butanol in an exposure chamber and four hours after exposure termination. A total of 19 cytokines and chemokines were quantified. Furthermore, at baseline and during the exposure session, participants rated the perceived intensity, valence and levels of symptoms and autonomic recordings were obtained.

**Results:**

The physiological and psychophysical measurements during the *n*-butanol exposure session verified a specific response in MCS individuals only. However, MCS subjects and healthy controls displayed similar upper airway inflammatory mediator profiles (P>0.05) at baseline. Likewise, direct comparison of mediator levels in the MCS group and controls after *n*-butanol exposure revealed no significant group differences.

**Conclusion:**

We demonstrate no abnormal upper airway inflammatory mediator levels in MCS subjects before or after a symptom-eliciting exposure to low dose *n*-butanol, implying that upper airways of MCS subjects are functionally intact at the level of cytokine and chemokine production and secretory capacity. This suggests that previous findings of increased cytokine plasma levels in MCS are unlikely to be caused by systemic priming via excessive upper airway inflammatory processes.

## Introduction

A substantial proportion of the adult population report different degrees of chemical intolerances towards everyday chemicals, e.g. fragranced consumer products, car exhaust, pesticides and new furniture’s at concentrations usually considered to be harmless [1]. The estimated prevalence varies substantially but in about 0.5% to 6.3% of the population [2–4,4–8], exposures to everyday chemicals elicit a complex array of symptoms at a much greater magnitude and often with disabling consequences in the form of social and occupational lifestyle changes and reduction in life quality [9,10]. Multiple chemical sensitivity (MCS) is a common term used to describe this severe form of chemical intolerance [11,12].

Controversially, no dose-response relationship has been identified linking exposure concentration to the symptom magnitude in MCS [13] and the type and severity of reported symptoms to an exposure are highly variable. Symptoms from the central nervous system (CNS) conveyed by migraine headaches, dizziness, extreme fatigue, and concentration difficulties are common, often combined with non-specific symptoms from other organ systems such as the mucosa/respiratory tract, musculoskeletal system and/or the gastro-intestinal tract [8,11,13].

Both physiological and psychological processes have been suggested as underlying mechanisms in MCS, but no definit conclusions can be drawn from available studies at this point. Dysregulation of the immune system and/or neurogenic inflammation is frequently proposed as mechanisms likely to play a role in the disease aetiology [14–21] and it has thus been suggested that immunological mediators might play a role in linking the immune and neural systems in these disorders, thereby playing a pivotal role in symptom elicitation [20,22–24]. Recently, altered levels of immunological mediators such as cytokines, chemokines and growth factors have been reported in blood plasma from subjects with MCS in two independent studies [14,20], however, the mechanism (s) or event(s) responsible for the abnormal mediator levels are unknown. It has been implied that MCS, at least partly, is a respiratory-based disorder involving excessive activation of the immune system in the upper airways, possibly due to induction of non-specific immune responses in the respiratory mucosa to low levels of irritants (airborne chemicals, particles or infectious agents) [15,25,26]. Immunological mediators secreted into the respiratory mucosa could subsequently be transferred directly into peripheral circulation affecting the mediator profile in the peripheral blood. Alternatively, is it possible that local inflammation in the upper airways can stimulate release of inflammatory mediators at a secondary location if sensory impulses from the original site of inflammation are being rerouted via the central nervous system, thereby causing neurogenic inflammation at a distant tissue site [21,27]. Evidence supporting this chain of events was provided by Kimata (2004) reporting increased plasma levels of inflammatory biomarkers in MCS subjects immediately after being exposed to a symptom eliciting mixture of volatile organic compounds while residing in a room that had recently been painted [28].

With focus on the inflammatory environment in the upper respiratory system, the aim of this study was to compare levels of inflammatory mediators at baseline in subjects with MCS versus an age- and gender-matched healthy control group and sequentially examine if exposure to low concentrations of the odorant *n*-butanol would convey a different upper airway inflammatory response in MCS subjects. In the attempt to mimic an authentic everyday experience described by subjects with MCS, the exposure was carried-out in an exposure chamber with full-body exposure. The inflammatory environment in the upper airways was monitored before and twice after the exposure session by measuring levels of selected cytokines and chemokines with different effector functions.

## Material and Methods

### Study population

Participants suffering from MCS and healthy controls were recruited through advertisement at public places and in a local Swedish newspaper. Exclusion criteria for participants of both groups included smoking, pregnancy, current breast feeding and doctor’s diagnosed fibromyalgia, chronic fatigue or irritable bowel syndrome. An additional exclusion criteria included anosmia and all participants were prior to the exposure screened for this condition using a 0.44% v/v (336 ppm) concentration of *n*-butanol (99%, Merck) of the Connecticut Chemosensory Clinical Research Center Threshold Test [29].

A total of 36 subjects who considered themselves especially sensitive were contacted by phone and pre-screened for eligibility using the US Consensus Criteria for MCS and the revisions suggested by Lacour et al. [11,12], which were operationalized as follows: 1) symptoms for at least 6 months; 2) symptoms occur in response to exposure to low-levels of chemicals that do not induce symptoms in other subjects who are exposed to the same levels; 3) symptoms occur when exposed and lessen or resolve when the symptom-triggering exposure is removed; 4) symptoms are elicited by at least two unrelated chemical substances; 5) presence of at least one symptom from the CNS (e.g. headache, fatigue, dizziness, memory problems, concentration difficulties or tiredness) and one symptom from another organ system; 6) symptoms cause significant impairment in daily life, either in social, recreational, occupational, educational, or economic situations (confirmed by the Chemical Sensitivity Scale-score in Table 1). A total of 18 subjects (16 women, 2 men) fulfilled the MCS criteria and were included in the study. Eighteen subjects (14 women, 4 men) were recruited as age- and sex-matched healthy controls. The controls did not fulfill any of the study criteria for MCS, and reported no avoidance behavior, annoyance or symptoms attributed to low-level chemical exposure. None of the participants in the control group shared housing with an MCS affected individual or had any close relative, i.e. parent, grandparent, sibling or child with MCS.

**Table 1:** Characteristics of the multiple chemical sensitivity (MCS) and the control group. Symptom Checklist (SCL).

	MCS group (n = 18)	Control group (n = 18)	*P-value* ^1^
Sex male/female, n	2/16	4/14	
Age mean (±SD)	44 (14)	41 (14)	.562
Chemical Sensitivity Scale mean (±SD)	96 (16)	72 (10)	**< .001**
Perceived Stress Scale, mean (±SD)	18 (6)	16 (8)	.522
SCL-90 Anxiety, mean (±SD)	0.5 (0.5)	0.5 (0.6)	.710
SCL-90 Depression, mean (±SD)	0.7 (0.6)	0.6 (0.7)	.743
SCL-90 Somatization, mean (±SD)	0.8 (0.6)	0.4 (0.4)	**.016**
Somatosensory Amplification Scale, mean (±SD)	29 (7)	25 (6)	.078

^1^ *p*-values refer to results of Mann-Whitney U-test.

### Demographic information and self-reported problems

Prior to exposure, background characteristics in terms of sex, age, morbidities, smoking or use of snuff were collected from all study participants and they were asked to fill in the following questionnaire instruments: (1) the Chemical Sensitivity Scale [30] to assess affective and behavioral reactions to everyday chemical exposure; (2) the anxiety, depression and somatization subscales of the Symptom Checklist-90 (SCL-90) inventory (Fridell et al., 2002; Derogatis et al., 1976); (3) the Perceived Stress Scale [31,32] to assess current levels of perceived stress; (4) the Somatosensory Amplification Scale [33] to assess to what degree respondents are bothered by uncomfortable visceral and somatic sensations.

### Exposure chamber and exposure procedure

Participants were exposed to *n-*butanol (99.4% J.T. Baker) while seated in a windowed exposure chamber. The exposure chamber had a volume of 2.7 m^3^ (height: 200 cm, width: 90 cm, depth: 150 cm) and the exposure concentration of *n-*butanol was 11.5 mg/m^3^ (3.7 ppm). The procedure is described in more details in Andersson et al. 2013 and Andersson et al. 2015. The odorant *n-*butanol was chosen for the exposure procedure based on a pilot test in which MCS sufferers judged the compound to be symptom-eliciting and because it had been used successfully in previous challenge studies [34,35].The concentration of *n-*butanol was clearly detectable (above the olfactory threshold 0.012mg/m^3^; [36]), but well below its threshold for sensory irritation (75mg/m^3^; [37]).

**Fig 1:**
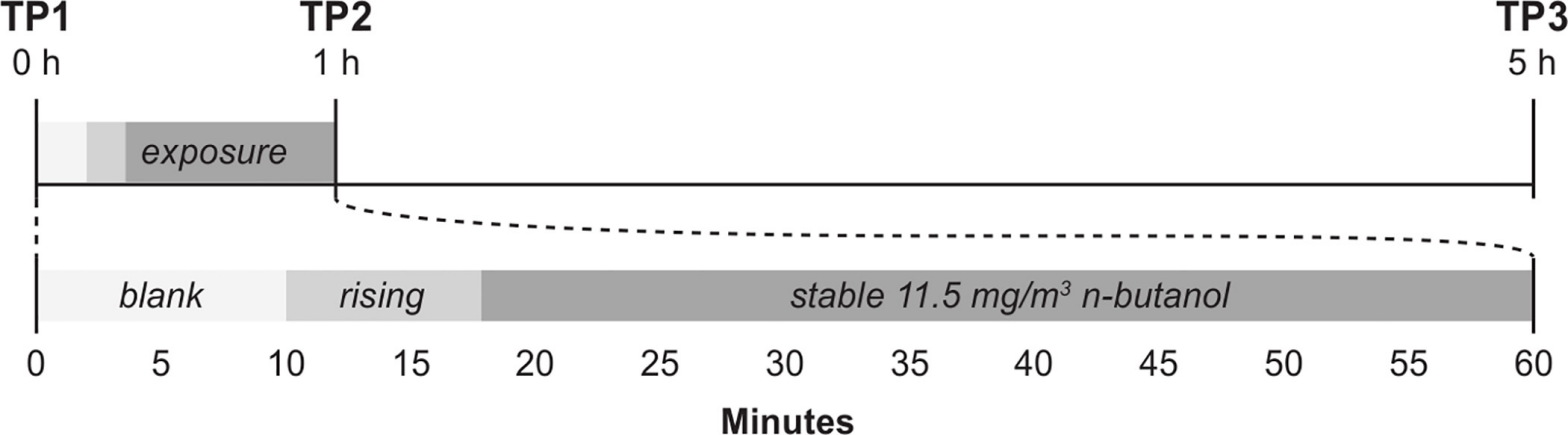
Overview of the exposure procedure and samples collection. During the first 10 min of testing, no odorant was delivered to the chamber, where after *n-*butanol was released into the chamber and reached its peak concentration about 8 min later. The concentration remained at this peak level (3.7 ppm) for the rest of the exposure session. Airway epithelial lining fluid was collected within 30 minutes prior to the exposure session (time point 1, TP1), within 15 minutes post exposure session (TP2) and again 4 hours after the exposure session was terminated (TP3).

Unknown to the participants, no odorant was delivered into the exposure chamber during the first 10 minutes of testing. After this initial period of blank exposure, *n-*butanol was released into the chamber and reached its peak concentration about 8 minutes later, as depicted in Fig 1. The concentration remained at this peak level for the rest of the session (42 minutes). The temperature and relative humidity inside the chamber was continuously monitored during the exposures and the mean temperature was 22°C (±1°C) and the relative humidity was 16% (± 2%) [38].

At baseline and at regular intervals during the exposure session, the participants rated the perceived intensity and valence as well as the levels of possible symptoms. Additionally, autonomic recordings of the breathing rate, tonic electrodermal activity, pulse rate and pulse rate variability were obtained at baseline and periodically during the exposure. A detailed description of the methodology used for collection of self-reported ratings and autonomic recordings has been published separately in Andersson et al. (2015), verifying the experimental setups ability to elicit characteristic MCS symptoms in the MCS group and to produce the expected group differences [38].

### Sample collection

Airway epithelial lining fluid was collected within 30 minutes prior to the exposure session (time point 1, TP1), within 15 minutes post exposure session (TP2) and again 4 hours after the exposure session had been terminated (TP3) as outlined in Fig 1. The participants could choose themselves where to stay and what to do during the period between TP2 and TP3. Sample material was collected using a recently introduced method that is non-invasive, easy manageable and highly sensitive [39]. **S**trips of 5 x 30mm filter paper were inserted gently by means of direct vision into the nasal cavity, laterally against the anterior part of the inferior turbinate. After insertion, a nasal clip was applied and the strips were left for absorption for 2 minutes [39–41]. Next, the strips were removed, placed in an Eppendorph tube and immediately stored at -80°C until analysis. Sample material was collected from both nostrils simultaneously. The filter material is composed of an absorptive synthetic hydroxylated polyester medium (Accuwik Ultra, fibrous hydroxylatedpolyester sheets, cat. no.SPR0730; Pall Life Sciences, Portsmouth, Hampshire, UK) optimized for collection, storage and conjugated release of sample material, and have successfully been utilized in studies with protein containing samples such as epithelial lining fluid [39–41].

### Approval

All research reported here was approved by the Umeå Regional Ethics Board, Sweden (Dnr 2013-19-31) and conducted in accordance with the Helsinki Declaration. All participants were given written and verbal information about the study, and signed informed consent was obtained from all study participants. All participants were given 500 SEK (~50 EUR) for their participation.

### Protein isolation

Total protein fractions were extracted from the filter papers by submerging the strips from each nostril into the cup of a cellulose acetate tube filter (0.22 μm pore size) within an Eppendorf tube (Spin-X Centrifuge Tube Filter, Sigma-Aldrich, Cat no.CLS8161), and applying 300 uL of Milliplex Assay Buffer (Millipore, Cat no. L-AB) added with 1x complete protease inhibitor cocktail (Roche Applied Science, Indianapolis, IN). The moist filter papers and assay buffer were then centrifuged for 5 minutes in a cooled centrifuge at 16000g. The tube containing extracted proteins from epithelial lining fluid was stored at -80°C until analysis.

### Quantification of cytokines and chemokines in epithelial lining fluid

We selected 19 cytokines and chemokines to represent inflammatory mediators associated with different type of immune responses. For simplicity, we grouped these mediators into categories representing type-1 (Th1/CD8+/NK cells/innate lymphoid cells (ILC), type-2 (Th2, eosinophils, ILC2), type-17 (Th17, neutrophils, ILC17), regulatory type (Treg) responses or lymphocyte activation properties based on the current perception on which cell types that produce the given mediators and/or are mainly affected by these mediators. Levels of the 19 immune mediators were measured in duplicates in the eluted samples using the Multi-Spot Human TH1/TH2 10-Plex Cytokine Assay System [interferon-γ (IFN-γ), interleukin-1β (IL-1β), IL-2, IL-4, IL-5, Chemokine (C-X-C motif) ligand 8 (CXCL8) /IL-8, IL-10, IL-12p70, IL-13 and tumor necrosis factor-α (TFN-α)], the Multi-Spot Human Chemokine 9-plex Assay System [CCL11/eotaxin-1, Chemokine (C-C motif) ligand 4 (CCL4)/macrophage inflammatory protein-1β, CCL26/eotaxin-3, CCL17/thymus activation-regulated chemokine, CXCL10/IFN-inducible protein-10, CXCL8/IL8, CCL2/monocyte chemotactic protein-1 (MCP-1), CCL22/ macrophage-derived chemokine and CCL13/MCP-4] and Human IL-17A Singleplex Assay (Meso Scale Discovery, Gaithersburg, MD). Plates were read at a Sector Imager 2400A (Meso Scale Discovery). The lower limit of detection for each mediator can be found in S1 Table.

### Total protein quantification

Total amount of protein in the eluted epithelial lining fluid was measured using the Qubit® Protein Assay Kits and a Qubit® 2.0 Fluorometer according to the manufacturer. Total protein concentrations in the 108 samples (36 x 3 TP) ranged from 3.20 to 4.98 mg/mL and cytokine and chemokine levels were normalized according to the total protein concentration in the samples as pg analyte/mg total protein.

### Statistical analyses

The SPSS 19.0 software package was used for statistical analysis; the level of significance was set at 0.05. Descriptive statistics for the two groups were generated and significant group differences were identified using independent samples t-tests. Untransformed as well as transformed cytokine and chemokine levels were examined for a normal distribution using a Shapiro-Wilk test and by visual inspection of histograms. Most variables showing a slightly skewed distribution continued to be skewed after log transformation, and all statistical analysis was carried out on the untransformed data.

Differences in cytokine and chemokine concentrations between the MCS group and the control group were examined using Mann–Whitney *U* test with the 19 cytokine and chemokine measurements as the dependent variables and the grouping variable, i.e. MCS or control, as the explanatory variable. A secondary outcome measure was differences over time within the MCS or the control group comparing mediator levels between the three TP´s. This test was performed using an independent-sample Kruskal–Wallis one-way analysis of variance with the 19 mediator measurements as the dependent variables and the three TP´s for collection of epithelial lining fluid as the explanatory variables. When statistically significant, Dunn's multiple comparisons procedure and Bonferroni correction was used to determine pairwise differences. Principal component analysis (PCA) was performed on combined normalized cytokine and chemokine values at TP1 using the software package Latentix 2.12 (www.latentix.com), and scores for principal component (PC) 1 and 2 were plotted against each other to detect combined immunological cytokine patterns in the dataset.

## Results

### Participant characteristics

Characteristics of the MCS and healthy control groups are shown in Table 1. A total of 36 subjects participated in the study; 18 fulfilling the criteria for MCS and 18 enrolled as healthy controls. Mean age was comparable between the two groups, while more women than men participated in the study. Compared to the controls, the MCS group showed a significantly higher score on the Chemical Sensitivity Scale (P < .001) and on the somatization subscale of the SCL-90 (P < .05). The MCS group also reported higher numbers of morbidities other than MCS [38]. No other significant demographic group differences were found.

### Soluble immune mediators within upper airways

Epithelial lining fluid was successfully collected from all 36 participants at the three sampling time points (Fig 1), and cytokine and chemokine levels were quantified in a total of 108 samples. To ascertain that data were not influenced by unexpected dilution due to differences in levels of secreted fluid volumes, we adjusted all immune mediator data based on the individual total protein content (ng/mL) per TP. The median concentration and interquartile range (IQR) measured are presented in Table 2 for each cytokine and chemokine at TP1, TP2 and TP3. The number and percentages of samples with cytokine or chemokine concentrations below limit of detection are presented in S1 Table.

**Table 2:** Quantified levels of immune mediators in epithelial lining fluid of upper airways. Median level with interquartile range of each mediator (in pg analyte/mg total protein) measured in the upper airway epithelial lining fluid from the MCS group and from the control group at the three time points (TP); TP1 at zero hours, TP2 at the end of exposure and TP3 4 hours post exposure session. Asterisks refer to significant differences (P ≤ 0.05) in analyte concentration between TP1, TP2 and TP3 within either the MCS or the control group identified using an Kruskal-Wallis test and Dunn's (1964) procedure with a Bonferroni correction for multiple comparisons as post-hoc analysis. CCL = Chemokine (C-C motif) ligand, IL = interleukin, IFN-γ = interferon-γ; TNF-α = tumor necrosis factor-α. The immunological mediators are sorted according to whether they are mainly represented in type-1 responses, type-2 responses, type-17 responses or involved in immune regulation (Reg) or activation (Act).

		MCS group (n = 18)			Control group (n = 18)		
		TP1	TP2	TP3	TP1	TP2	TP3
**Type 1**	IFN-γ	4.48 (2.01–6.85)	4.72 (1.87–6.63)	1.93 (0.70–3.11)*	3.93 (3.13–7.63)	4.63 (1.68–6.23)	2.19 (0.70–3.07)*
	IL-12p70	8.16 (6.00–19.10)	3.82 (0.82–10.84)	4.12 (0.62–16.73)	8.53 (5.09–15.83)	5.07 (2.45–19.12)	4.46 (1.97–9.92)
	CXCL10	4957 (2255–8405)	2942 (1350–6853)	5752 (3497–9911)	5778 (2325–7123)	5469 (1318–7815)	3913 (1931–5796)
	TNF-α	83.03 (32.50–221.3)	60.09 (23.30–132.9)	42.93 (14.77–212.5)	82.65 (44.23–231.8)	47.94 (18.40–132.6)	63.62 (21.05–104.1)
	CCL2	567.6 (337.8–567.6)	423.8 (220.2–752.5)	604.8 (324.3–1045)	358.6 (274.7–751.1)	437.3 (187.7–640.6)	316.5 (238.5–748.4)
	CCL4	228.0 (82.65–472.5)	169.7 (75.33–276.9)	112.7 (44.41–332.1)	205.8 (135.9–387.6)	182.2 (105.4–282.1)	143.6 (85.6–248.2)
**Type 2**	IL-4	1.98 (0.34–4.15)	1.99 (0.85–3.33)	0.003 (0.002–0.89)*	1.51 (055–4.31)	1.48 (0.85–2.56)	0.003 (0.003–24.02)*
	IL-5	11.23 (4.51–20.48)	4.7 (2.99–15.2)	5.40 (3.32–13.4)	8.43 (3.77–19.53)	10.13 (2.80–18.71)	5.30 (2.62–12.39)
	IL-13	95.56 (47.75–185.4)	74.54 (21.98–127.6)	40.59 (24.79–182.8)	98.36 (51.72–161.1)	61.91 (31.51–110.8)	49.15 (17.36–125.1)
	CCL11	607.3 (310.3–1047)	525.7 (224.1–867.8)	621.9 (313.5–1064)	522.4 (361.4–1108)	535.5 (254.7–840.4)	376.8 (180.0–974.7)
	CCL13	47.98 (37.30–57.73)	42.69 (29.01–59.87)	29.86 (16.21–51.17)	38.74 (31.09–53.03)	42.03 (28.66–51.27)	32.24 (22.16–48.12)
	CCL17	31.26 (17.25–49.97)	28.62 (16.3–33.94)	25.63 (16.87–60.30)	26.95 (12.53–51.68)	28.30 (13.37–49.18)	18.11 (12.0–35.76)
	CCL22	953.0 (724–1262)	955.8 (761.1–1202)	513.8 (94.66–1031)*	949.8 (855.5–1068)	998.0 (791.4–1205)	701.5 (410.6–969.9)*
	CCL26	71.84 (43.02–123.5)	81.11 (40.47–104.2)	0.07 (0.06–75.16)*	73.10 (36.56–133.5)	85.11 (43.08–183.9)	0.07 (0.06–24.02)*
**Type 17**	IL-1β	132.4 (52.35–318.7)	73.05 (29.30–116.3)	70.08 (26.61–157.9)	222.7 (90.45–322.4)*	83.05 (38.24–122.2)	76.45 (49.07–125.0)
	IL-17A	0.51 (0.25–2.37)	0.35 (0.02–1.35)	0.37 (0.02–1.36)	1.88 (0.15–3.07)	0.49 (0.05–3.01)	1.20 (0.24–2.85)
	CXCL8	9835 (5268–14773)	6450 (2926–10199)	6085 (1899–9418)	7880 (4747–14866)	6699 (2732–8407)	5159 (3858–7529)
**Reg.**	IL-10	31.95 (19.34–49.73)	30.77 (11.98–56.25)	16.09 (10.23–41.10)	28.29 (13.85–42.33)	21.00 (12.56–31.68)	16.70 (12.51–29.05)
**Act.**	IL-2	123.2 (63.94–182.5)	55.78 (28.63–87.73)	49.25 (15.91–125.3)	88.50 (37.52–176.18)	48.83 (19.62–140.4)	33.68 (16.84–89.45)

### Baseline levels of upper airway mediators

**Fig 2:**
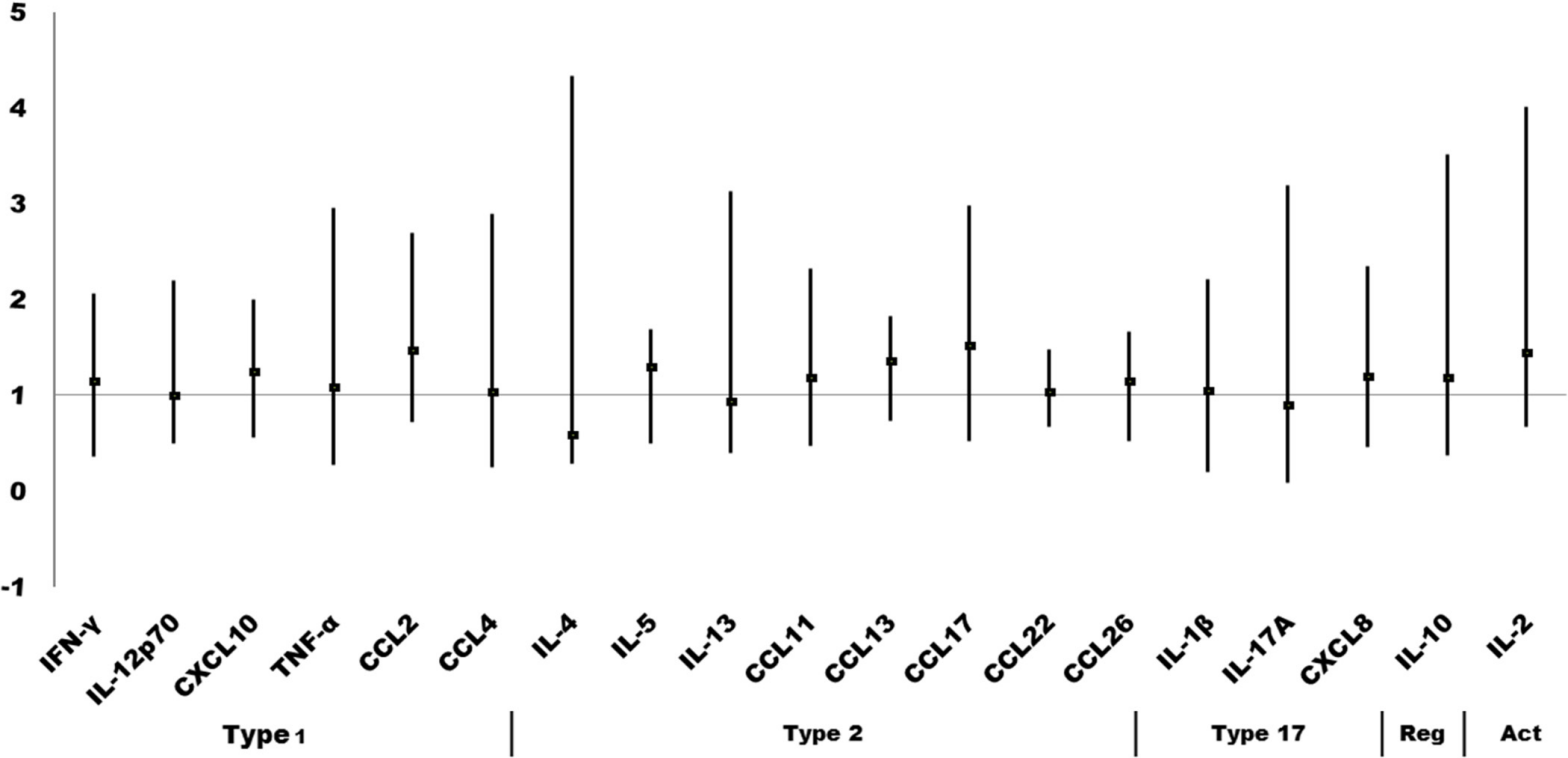
Ratios of upper airways inflammatory mediators in unexposed MCS versus healthy controls. Data represent ratios of medians with 95% confidence interval between cytokine and chemokine levels in the upper airway epithelial lining fluid in MCS subjects and healthy controls. CCL = Chemokine (C-C motif) ligand, IL = interleukin, IFN-γ = interferon-γ; TNF-α = tumor necrosis factor-α. The immunological mediators are sorted according to whether they are mainly represented in type-1 responses, type-2 responses, type-17 responses or involved in immune regulation (Reg) or activation (Act).

One primary objective was to identify differences in cytokine/chemokine regulation in upper airways between the MCS and the control group at baseline. However, no statistically significant group differences were identified in upper airway mediator levels at TP1 for any of the 19 mediators included (Fig 2). Moreover, using a multivariate principal component analysis to explore if the pattern of inflammatory markers differed in MCS as opposed to controls, we found no statistically significant differences (S2 Fig).

### Upper airway response profiles to *n*-butanol exposure

**Fig 3:**
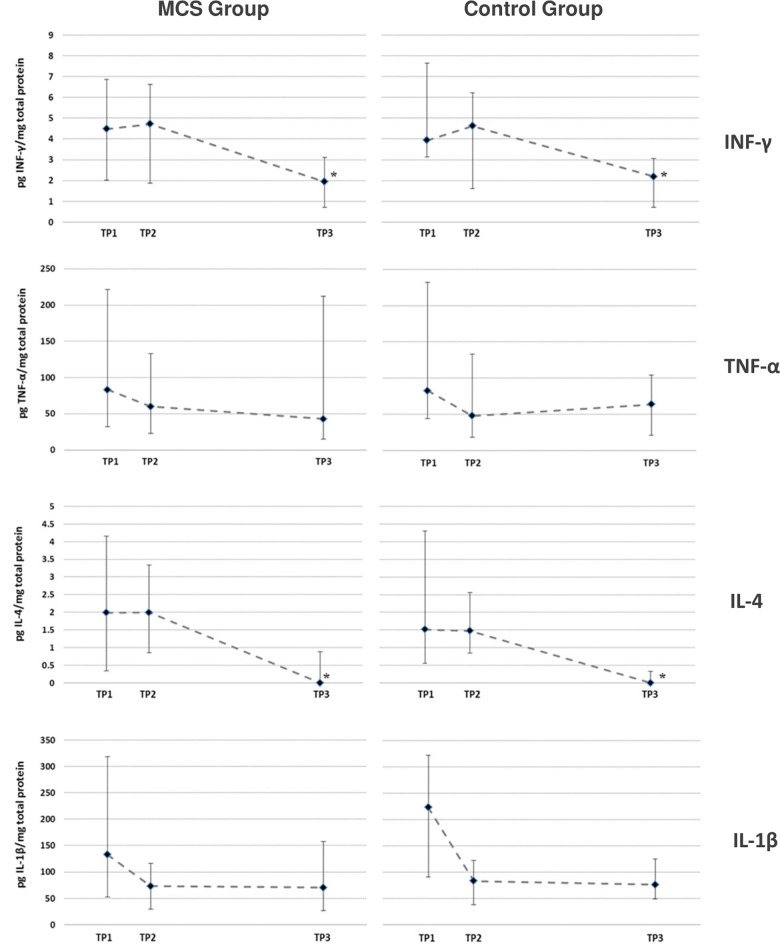
*n*-butanol exposure and upper airway inflammatory mediators. Data are shown as median level with interquartile range of interferon-γ (IFN-γ), tumor necrosis factor-α (TNF-α), Interleukin-4 (IL-4) and IL-1β (in pg analyte/mg total protein) measured in the upper airway epithelial lining fluid from the MCS group and from the control group at the three time points (TP); TP1 at time zero, TP2 at the end of the *n*-butanol exposure and TP3 4 hours post exposure. Asterisks refer to significant differences (P ≤ 0.05) in analyte concentrations between TP1, TP2 and TP3 within either the MCS or the control group.

MCS subjects perceived the *n*-butanol exposure as being more intense, more unpleasant and rated symptoms to be of greater magnitude compared to controls as reported in Andersson et al. (2015) [38]. Here we examined if the difference in symptom elicitation was associated with *in situ* changes in the release of upper airway inflammatory mediators. A direct comparison of mediator levels measured at TP2 and TP3 was performed between the two groups, since the similar baseline levels between MCS subjects and controls allowed for a direct comparison without prior adjustment. We identified no significant differences for any of the 19 cytokine and chemokines upon *n*-butanol exposure (Table 2). Median values with IQR for cytokines IFN-γ, TNF-α, IL-1β and IL-4 at each TP are presented in Fig 3 and similar data for the remaining 15 mediators are shown in S1 Fig.

### Time-course changes

The repeated sampling of epithelial lining fluid at the three consecutive time points allowed for analyses of time-course differences in MCS and controls. The comparison revealed several time-dependent changes in mediator concentrations (Fig 3, S1 Fig and Table 2). For IFN-γ, IL-4, CCL22 and CCL26, concentrations decreased significantly at TP3 compared to TP1 and TP2 in both groups. For IL-1β, the analysis showed a significantly higher level at TP1 compared to TP2 and TP3 in the control group but not in the MCS group. However, altogether we found no apparent group differences in secretion of immune mediators in upper airway epithelial lining fluid over the course of the study.

## Discussion

We examined whether local upper airway inflammatory responses were excessively activated in MCS subjects, thereby playing a role in symptom elicitation. A broad panel of 19 representative pro- and anti-inflammatory cyto- and chemokines were selected to be measured *in situ* within upper airways of MCS subjects and healthy controls at baseline and after a controlled low-dose *n*-butanol exposure shown to elicit symptoms in MCS subjects only [38]. Our data demonstrated no abnormal inflammatory mediator levels in the upper airways at any time point in MCS subjects. These findings suggest that upper airways of MCS subjects are functionally intact at the level of inflammatory mediator responsiveness and release.

The demographic data showed that the MCS group scored higher on the Chemical Sensitivity Scale suggesting that the case criteria for inclusion in the study were successful. As presented by Andersson et al. (2015), over the course of the exposure, the MCS group perceived the *n*-butanol exposure as being more intense, more unpleasant and rated symptoms to be of greater magnitude compared to controls [38]. Additionally, individuals with MCS had higher than normal pulse rate and lower than normal pulse rate variability [38], indicating abnormal regulation of the sympathetic branch of the autonomic nervous system during the exposure [42]. Accumulated, these findings verify the ability of the experimental setup to produce the expected symptom elicitation in MCS while not in healthy controls, and therefore provided a reliable platform for the upper airway immunological comparisons presented herein.

Immunological mediator profiles were assessed at baseline in both unexposed airways of asymptomatic MCS subjects and controls and in exposed airways from the same individuals. This study design made it possible to compare and infer systematic changes within the individual’s secretory capacity, as well as within the MCS and control group, providing an ideal comparative platform for detection of MCS-associated inflammatory regulation of *in situ* upper airway responses to the exposure.

Before initiating the study we made the assumption that for airway inflammation to be involved in the systemic fast response observed after chemical exposure in MCS subjects, any effector molecules of disease relevance would have to been synthesized, i.e. available, prior to or at the time for symptom elicitation. This is important as the normal protein processing machinery takes around 5–6 hours from cellular activation to synthesis and secretion of new proteins after activation of epithelial and immune cells, while symptom elicitation in MCS subjects most often takes place within minutes to an hour after contact with environmental triggering agents [43]. The reported instant chemically-induced response in MCS subjects was also apparent in the current study where the MCS group differed significantly (p < .001) from the control group on symptoms ratings after just 30 minutes exposure [38]. Based on this assumed necessity of immediate availability of inflammatory mediators in order to convey disease symptoms, we decided to collect all repeated samples within a short time frame after *n*-butanol exposure, and consequently, any changes in mediator levels over time would have to be due to secretion of already synthesized and constitutively deregulated levels of mediators from activated effector cells.

Our finding of similar cytokine and chemokine profiles in unexposed (baseline) upper airways of MCS and healthy individuals suggests that no abnormal inflammation or immune regulation is taking place in the upper airway of the MCS subjects during normal unexposed conditions. Moreover, our observation of similar cytokine and chemokine levels in upper airways of MCS subjects and controls after low-dose *n*-butanol exposure, despite MCS subjects experiencing a deviating reaction to the exposure session, implies that symptom elicitation in MCS takes place without localized secretion of immunological mediators from epithelial and immune cells in the upper airways. Likewise, our finding underpins that previous reports of elevated cytokine levels within circulating blood [14,20] is without involvement of upper airway inflammatory mediators being secreted and transferred into peripheral circulation.

Although no MCS-associated airway inflammatory responses could be linked to the exposure session, the time-course analysis revealed changes in mediator levels over time with IFN-γ, IL-4, CCL22 and CCL26 levels decreasing statistically significantly at TP3 compared to the earlier TP’s in both MCS and controls. For IL-1β, the data showed a significantly increased level at TP1 compared to TP2 and TP3 in the control group, with the same tendency for MCS. Since these time-dependent changes are not exclusive to one of the groups, we interpret that it cannot be linked to MCS, but might be attributed to a common regulatory mechanism, possibly as a result of a non-MCS associated response to the exposure chamber conditions. Alternatively, it could be a consequence of a methodically introduced dilution factor caused by repeated sampling with three samples collected within a five hours period. This phenomenon has been reported for sampling of epithelial lining fluid using nasal lavage, where it is recommended only to collect one sample daily [44]. However, we found no changes in the total level of protein (approx. 360 ug/mL protein per sample) at any time point, why the drop in some mediators from TP1 to TP3 seems unlikely to be due to an overall methodically introduced dilution factor. Rather it could relate to the short interval between sampling points, simply not allowing for re-synthesis of some cytokines and chemokines that therefore diminish due to low level secretion.

For this study we utilized a newly implemented filter paper-based method for collection of epithelial lining fluid onto an absorbent matrix introduced by Chawes et al. (2010) [39]. This method has the advantage that epithelial lining fluid is collected without disturbing the epithelial cell layer and without introducing a considerable and variable dilution factor [39], which is often the case using nasal lavage [45–48]. We found that this technique was easily implemented, inexpensive and provided reliable data, and except from a minor tickling sensation while having the filter pieces inserted, the participants reported no discomfort, hence making the technique ideal for repeated sampling of epithelial lining fluid. Moreover we decided to measure and adjust for total protein concentration with the epithelial lining fluid per individual at all TPs. This decision was made in order to account for possible inter-individual differences in fluid secretion capacities (e.g. due to runny nose).

It is a limitation of the study that the number of enrolled participants in each group is relatively low; however, 36 subjects was the number of participants that could be systematically handled within this study design of repeated measures and controlled *n*-butanol exposure. Symptomatic heterogeneity among the MCS individuals is a recurrent challenge for all clinical research within MCS, and although the inclusion criteria appears to be adequate based on group response [38] and MCS group characteristics, it is a possible that the experimental setup is unable to reveal group differences because of the relatively low number of participants and the considerable variation in response patterns observed between MCS subjects. However, since the variation within the healthy control group was observed to be at similar levels, it is less likely that heterogeneity within the MCS group is the direct cause of the study outcome.

It would also have strengthened the study if we had included a blank exposure sample in a blinded setup. This way it would have been possible to determine whether any immunological changes were purely a result of biological interactions between the exposure and responding cell types in upper airways, or if psychological mechanisms, like a stress response and the anticipated discomfort would have contributed to or been the principal mechanism responsible for the changes. Unfortunately, due to the design of the exposure chamber, it was not possible to perform dual exposures, and to collect epithelial lining fluid continuously during the course of the exposure. Furthermore, the aim of this study was to reveal if the exposure session would trigger differential secretion of immunological mediators in the upper airway of MCS subjects and not necessarily to identify any underlying causes of differences at this present stage.

## Conclusion

In conclusion, the results from this study suggests that a MCS symptom-eliciting exposure using a low dose of the odorant *n*-butanol does not trigger a local inflammatory response in upper airways based on cytokine and chemokine profiles. The systemic response to the exposure including differences in chemosensory perception is thereof suggested not to be initiated or influenced by upper airway inflammation. Based on these findings, it seems unlikely that the previously reported alterations in blood plasma cytokine levels in unexposed MCS subjects is caused by exaggerated upper airway inflammatory responses.

## Supporting Information

S1 Fig Time-dependent release of inflammatory mediators after n-butanol exposure. Data represents median level with interquartile range of interleukin-12p70 (IL-12p70), Chemokine (C-C motif) ligand 2 (CCL2), CCL4, CCL11, CCL13, CCL26, IL-5, IL-13, CCL7, CCL22, CXCL8, IL-17A, IL-10 and IL-2 (in pg analyte/mg total protein) measured in the upper airway epithelial lining fluid from the MCS group and from the control group at the three time points (TP); TP1 at time zero, TP2 at the end of *n*-butanol exposure and TP3 4 hours post exposure. Asterisks refer to significant differences (P ≤ 0.05) in analyte concentrations between TP1, TP2 and TP3 within either the MCS or the control group.
(PDF)

S2 Fig Principal component analysis showing no differences between MCS and controls at baseline. A, Combined loadings and scores plot for PC1 and PC2 at T1, where MCS individuals are colored by red and controls by blue, loadings are in black. B, Statistics on extracted PC1 and PC2 components for all individuals, subdivided into MCS and controls.
(DOCX)

S1 Table Numbers and percentages of samples with cytokine or chemokine concentrations below the assay limit of detection (LOD). The immunological mediators are sorted according to whether they are mainly involved in type-1 responses, type-2 responses, type-17 responses or in immune regulation (Reg) or activation (Act).
(DOCX)

S2 Table Minimal data set. Mediator concentrations (pg analyte/mg total protein) measured in all samples.
(XLSX)
