# Supplementary figures and images for: Inflammatory Mediator Profiling of n-butanol Exposed Upper Airways in Individuals with Multiple Chemical Sensitivity
Source: PLoS One. 2015 Nov 23;10(11):e0143534. doi: 10.1371/journal.pone.0143534 (PMC4657963; doi:10.1371/journal.pone.0143534)

## MCS Group

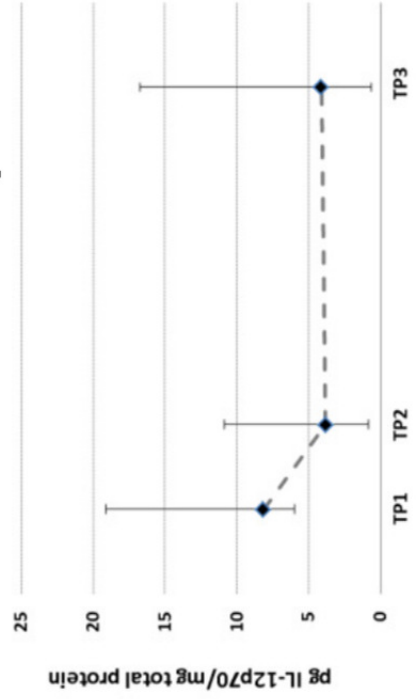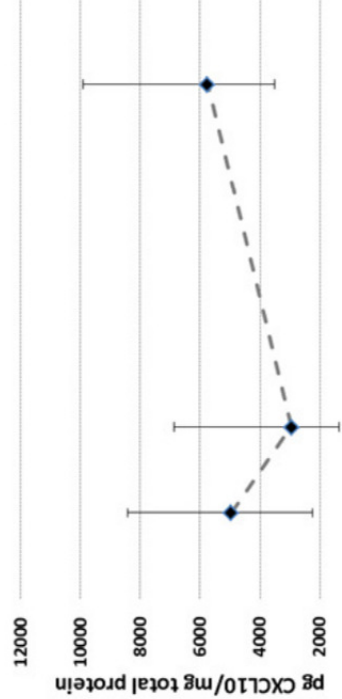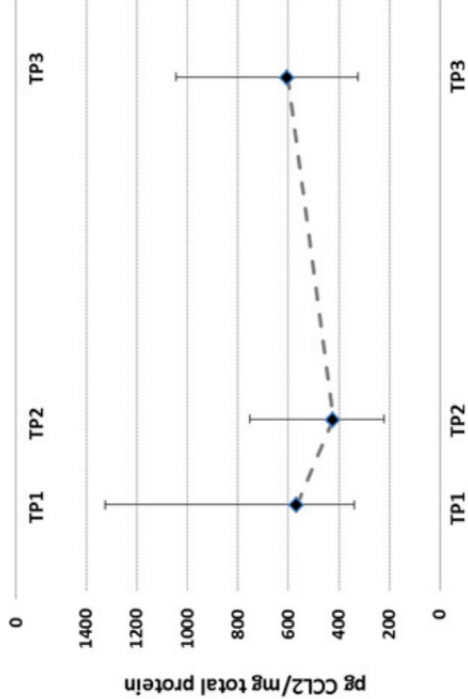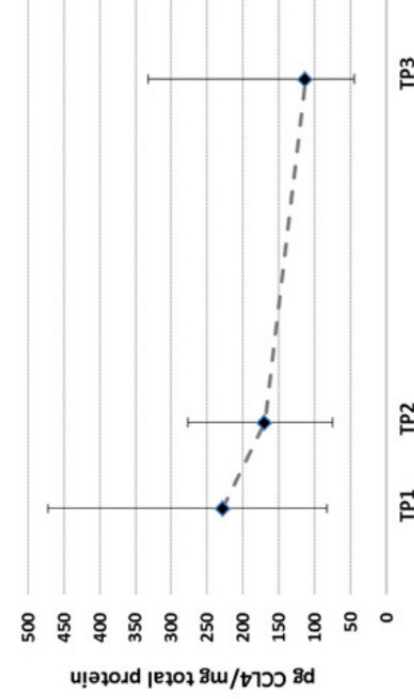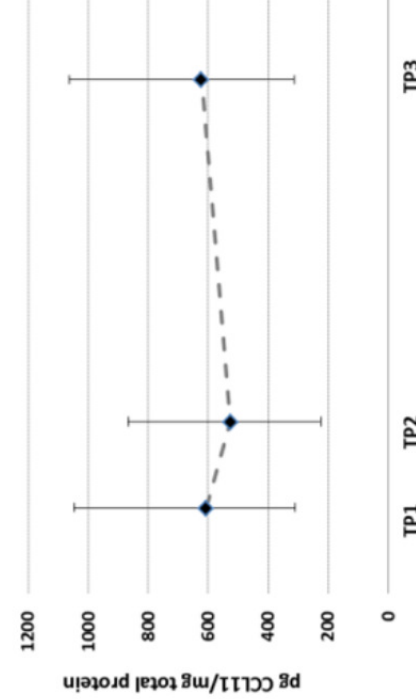

## Control Group

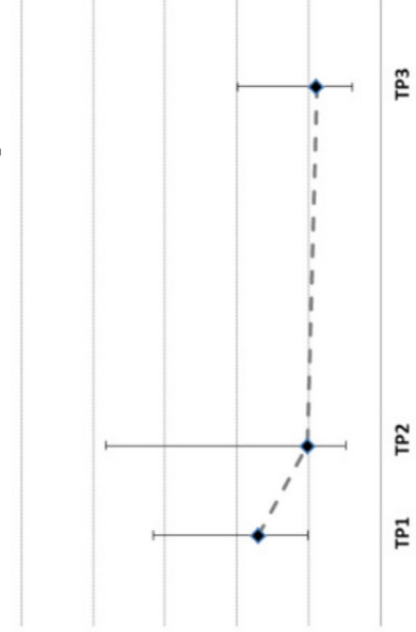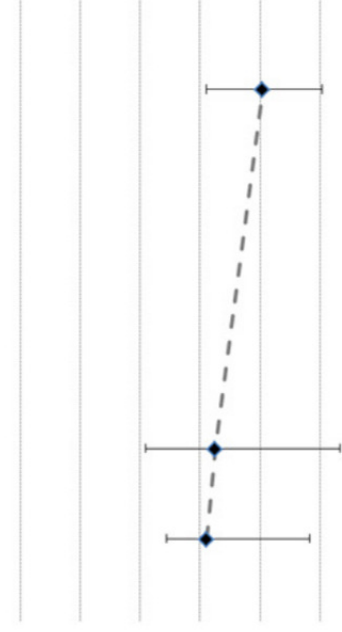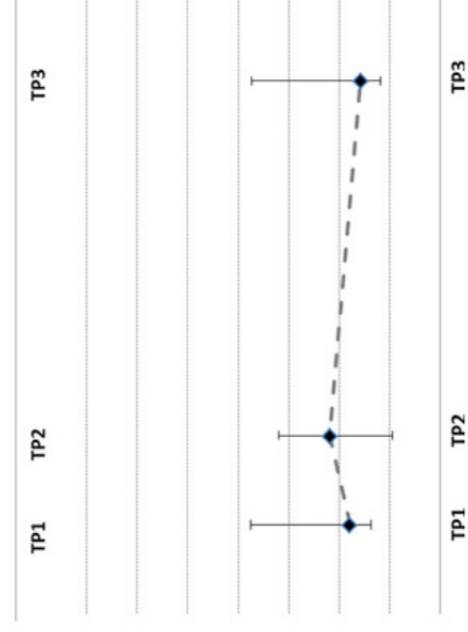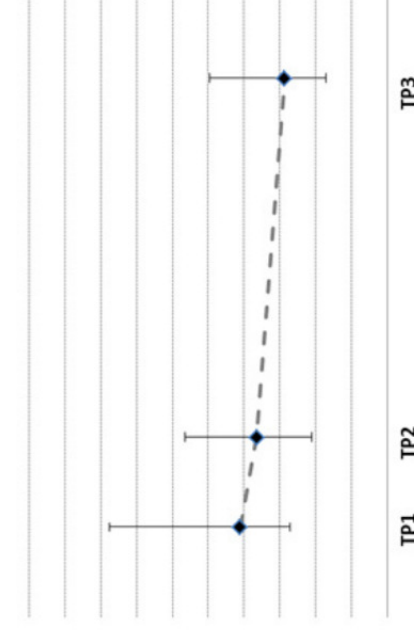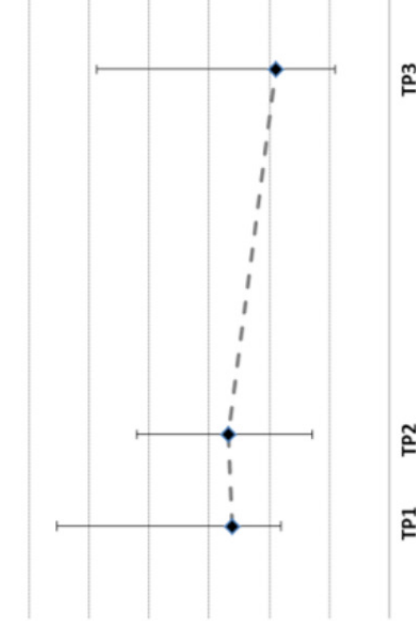

MCS Group

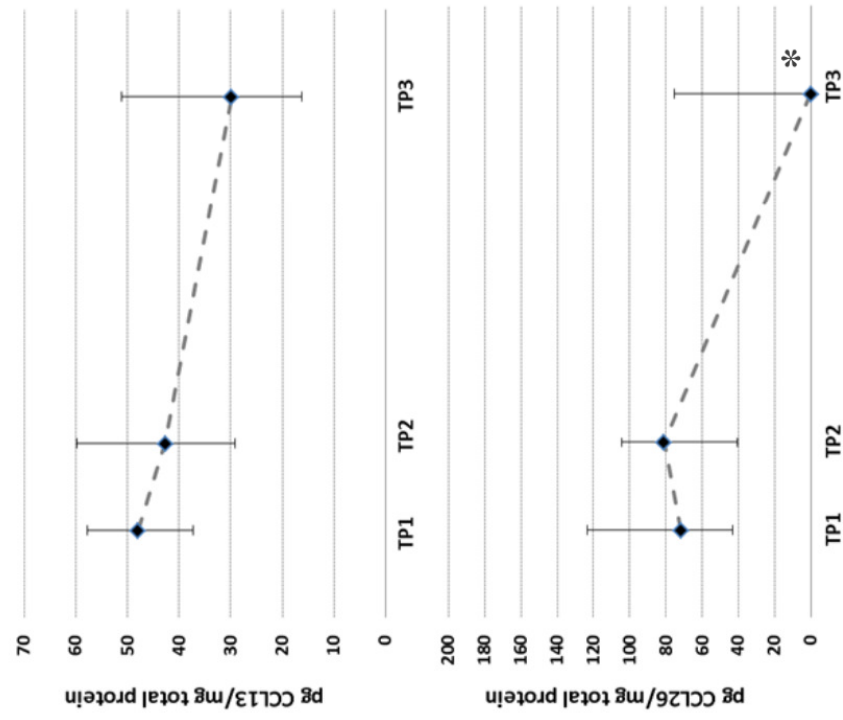

Control Group

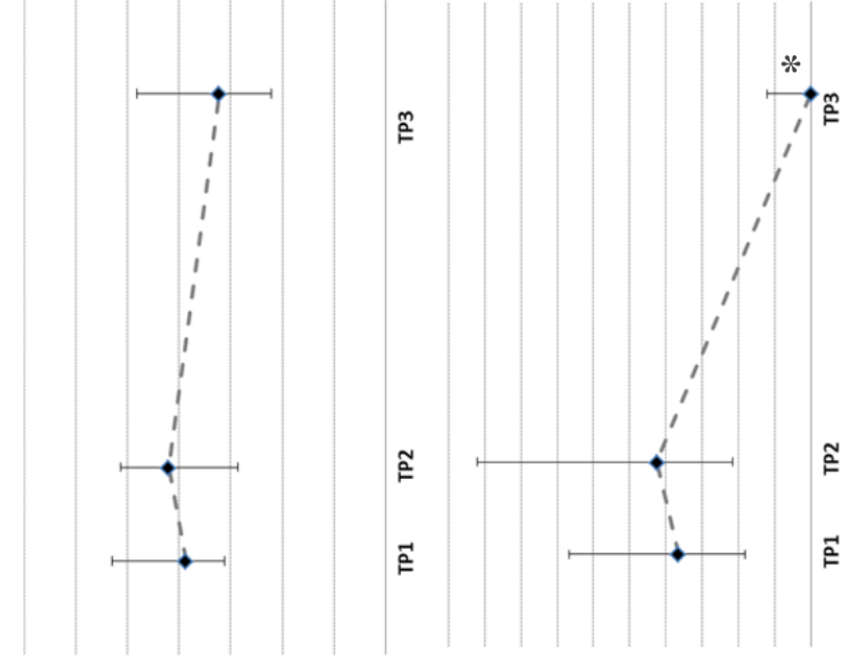

CCL26

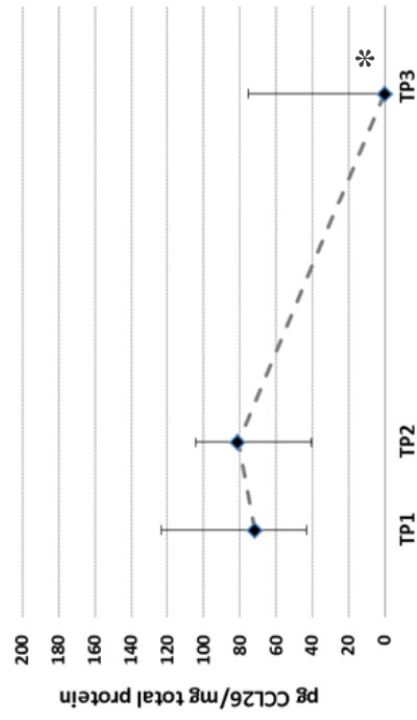

IL-5

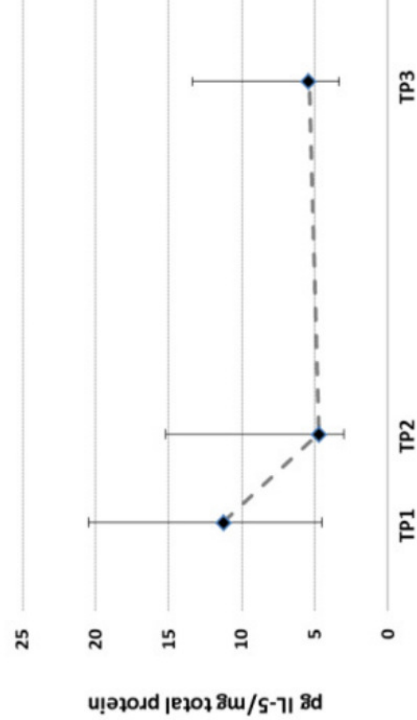

IL-13

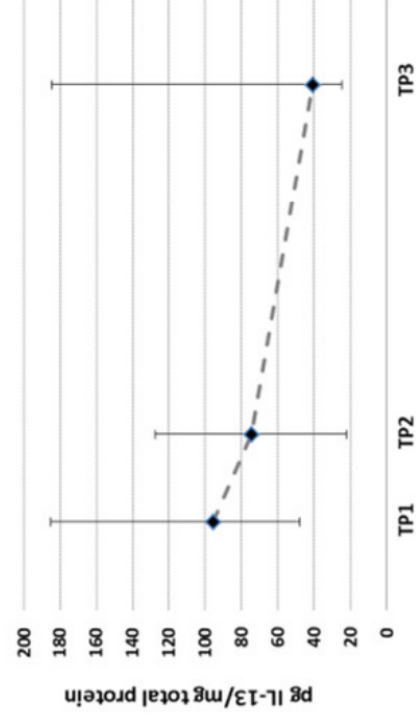

CCL17

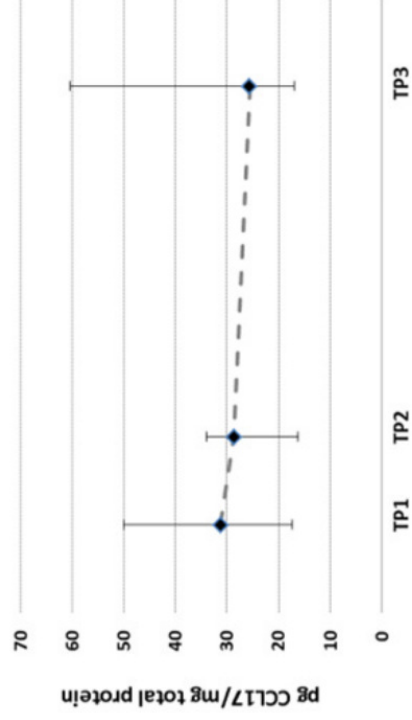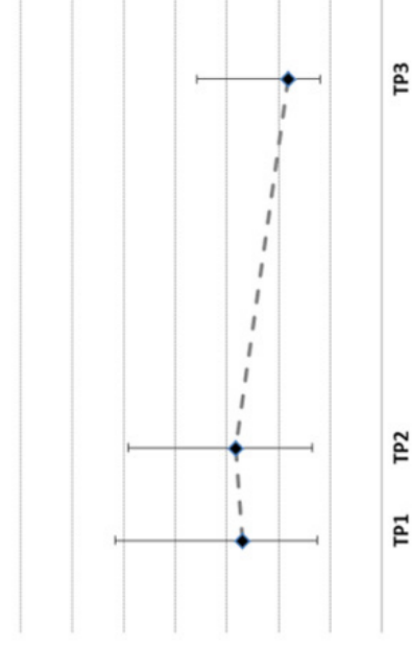

## MCS Group

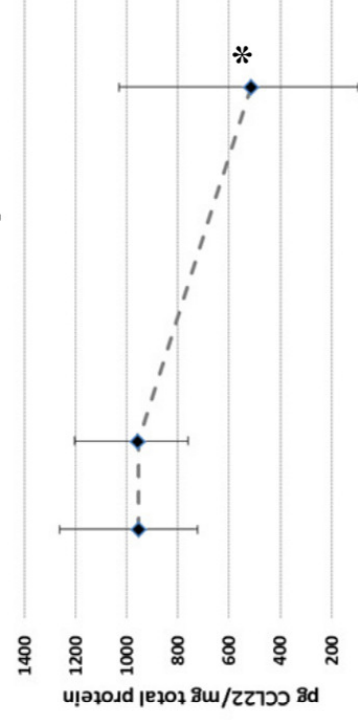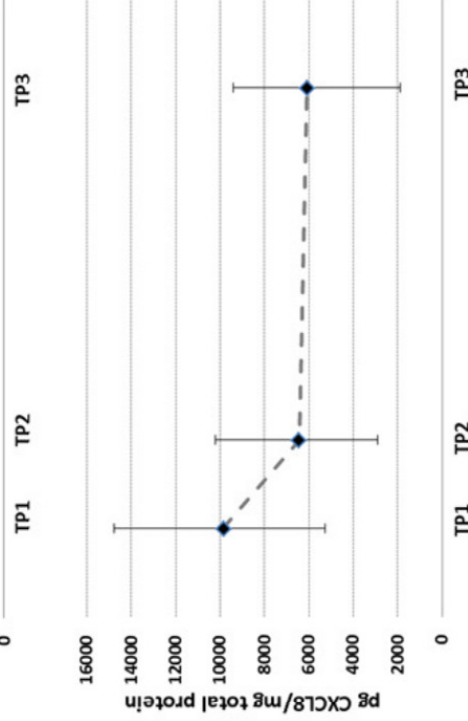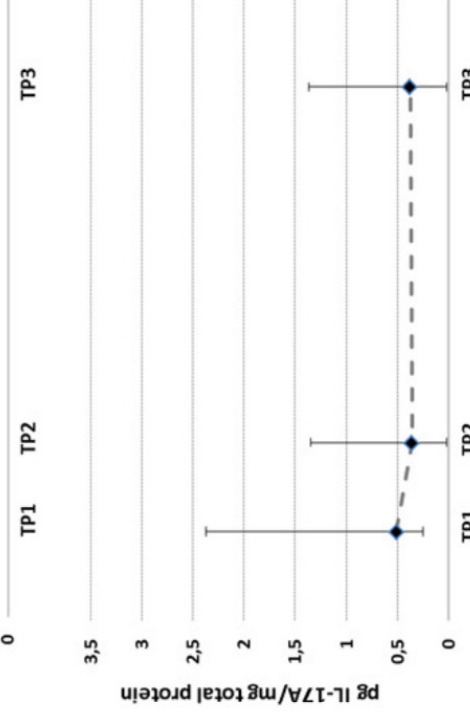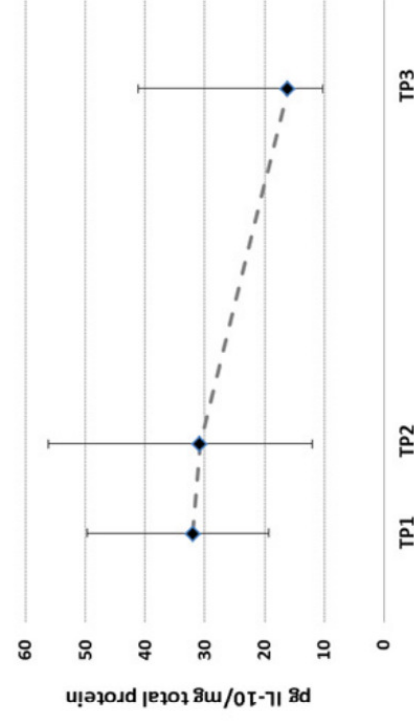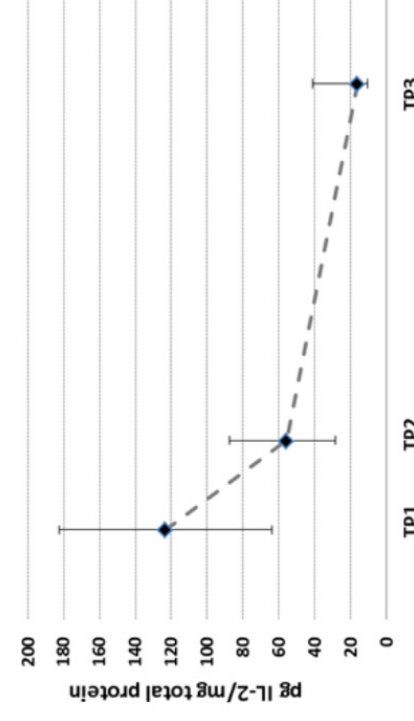

## Control Group

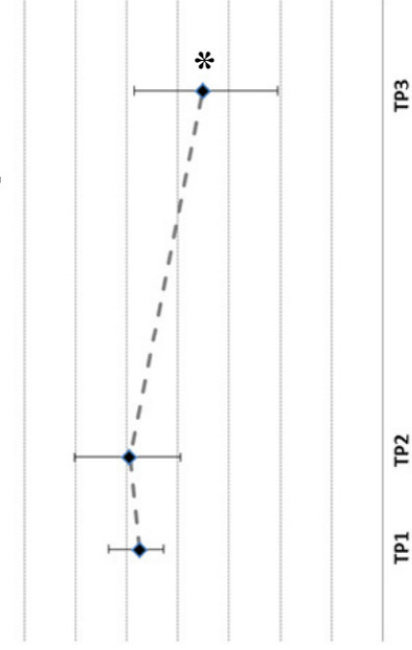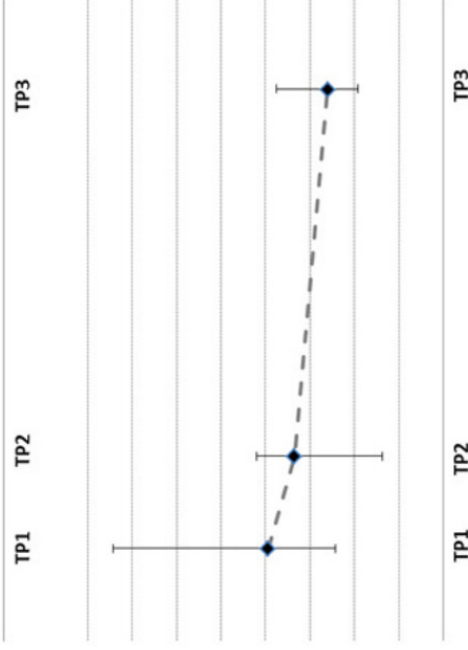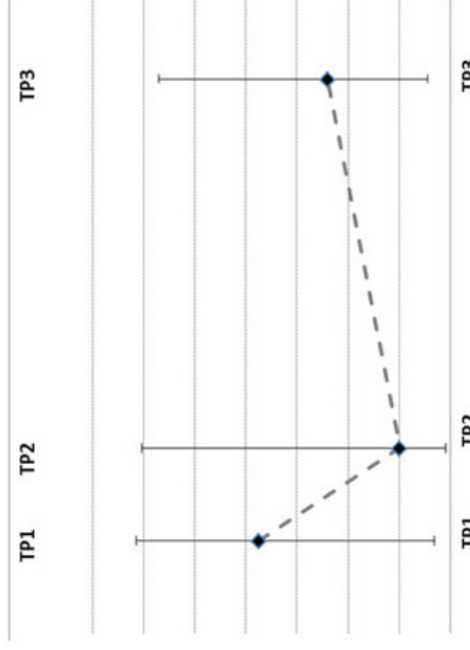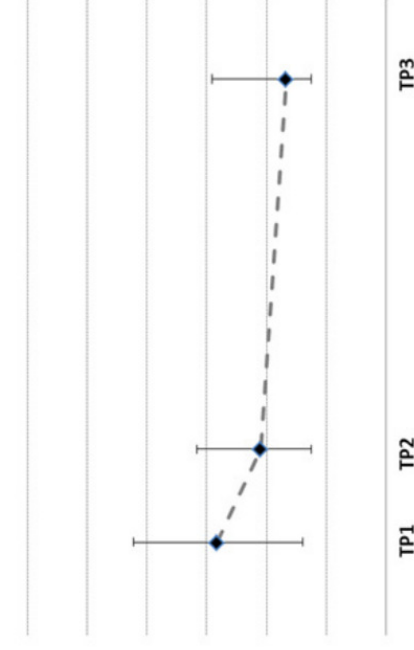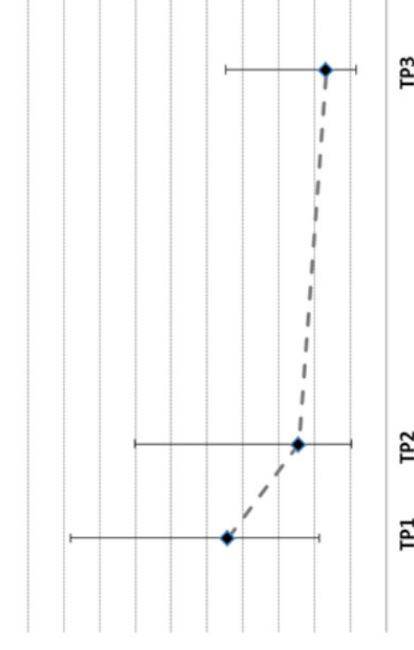

Supplement: S1 Fig — Data represents median level with interquartile range of interleukin-12p70 (IL-12p70), Chemokine (C-C motif) ligand 2 (CCL2), CCL4, CCL11, CCL13, CCL26, IL-5, IL-13, CCL7, CCL22, CXCL8, IL-17A, IL-10 and IL-2 (in pg analyte/mg total protein) measured in the upper airway epithelial lining fluid from the MCS group and from the control group at the three time points (TP); TP1 at time zero, TP2 at the end of n-butanol exposure and TP3 4 hours post exposure. Asterisks refer to significant differences (P ≤ 0.05) in analyte concentrations between TP1, TP2 and TP3 within either the MCS or the control group. (PDF) [file pone.0143534.s001.pdf]
