# Supplementary material for: Inflammatory Mediator Profiling of n-butanol Exposed Upper Airways in Individuals with Multiple Chemical Sensitivity
Source: PLoS One. 2015 Nov 23;10(11):e0143534. doi: 10.1371/journal.pone.0143534 (PMC4657963; doi:10.1371/journal.pone.0143534)
Supplement: S2 Fig — A, Combined loadings and scores plot for PC1 and PC2 at T1, where MCS individuals are colored by red and controls by blue, loadings are in black. B, Statistics on extracted PC1 and PC2 components for all individuals, subdivided into MCS and controls. (DOCX) [file pone.0143534.s002.docx]

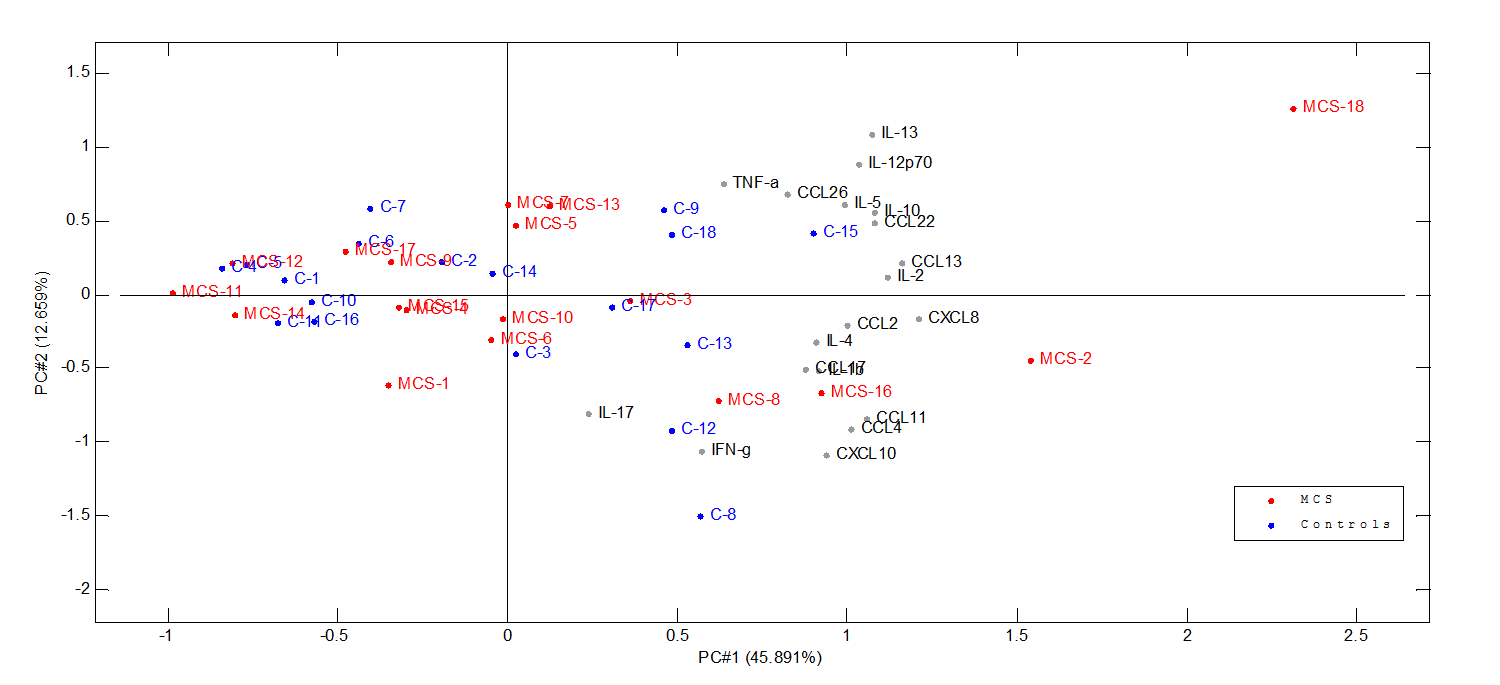


**A**

**B**

**S2 Fig: Principal component analysis showing no differences between MCS and controls at baseline**. A, Combined loadings and scores plot for PC1 and PC2 at T1, where MCS individuals are colored by red and controls by blue, loadings are in black. B, Statistics on extracted PC1 and PC2 components for all individuals, subdivided into MCS and controls.
