# Supplementary material for: Inflammatory Mediator Profiling of n-butanol Exposed Upper Airways in Individuals with Multiple Chemical Sensitivity
Source: PLoS One. 2015 Nov 23;10(11):e0143534. doi: 10.1371/journal.pone.0143534 (PMC4657963; doi:10.1371/journal.pone.0143534)
Supplement: S1 Table — The immunological mediators are sorted according to whether they are mainly involved in type-1 responses, type-2 responses, type-17 responses or in immune regulation (Reg) or activation (Act). (DOCX) [file pone.0143534.s003.docx]

S1 Table: Numbers and percentages of samples with cytokine or chemokine concentrations below the assay limit of detection (LOD). The immunological mediators are sorted according to whether they are mainly involved in type-1 responses, type-2 responses, type-17 responses or in immune regulation (Reg) or activation (Act).

| Response type | | Analyte | n | Undetectable n | Undetectable % | LOD pg/mL |
| --- | --- | --- | --- | --- | --- | --- |
| Type 1 | | IFN-γ | 108 | 7 | 6.5 | 0.547 |
| Type 1 | | IL-12p70 | 108 | 13 | 12 | 0.567 |
| Type 1 | | CXCL10 | 108 | 0 | 0 | 13 |
| Type 1 | | TNF-α | 108 | 1 | 0.9 | 0.593 |
| Type 1 | | CCL2 | 108 | 0 | 0 | 1.88 |
| Type 1 | | CCL4 | 108 | 2 | 1.9 | 7.63 |
| Type 2 | | IL-4 | 108 | 28 | 25.9 | 0.66 |
| Type 2 | | IL-5 | 108 | 1 | 0.9 | 0.166 |
| Type 2 | | IL-13 | 108 | 0 | 0 | 0.926 |
| Type 2 | | CCL11 | 108 | 0 | 0 | 29.7 |
| Type 2 | | CCL13 | 108 | 0 | 0 | 6 |
| Type 2 | | CCL17 | 108 | 9 | 8.3 | 7.41 |
| Type 2 | | CCL22 | 108 | 7 | 6.5 | 231 |
| Type 2 | | CCL26 | 108 | 26 | 24.1 | 15.2 |
| Type 17 | | IL-1β | 108 | 0 | 0 | 0.954 |
| Type 17 | | IL-17A | 108 | 20 | 18.5 | 0.34 |
| Type 17 | | CXCL8 | 108 | 0 | 0 | 0.454 |
| Regulatory | | IL-10 | 108 | 2 | 1.9 | 0.329 |
| Activation | | IL-2 | 108 | 0 | 0 | 1.23 |
|  |  | |  |  |  |  |
